# RETRACTED: Martínez-Revuelta et al. Numerical Study of a Solar Cell to Achieve the Highest InGaN Power Conversion Efficiency for the Whole In-Content Range. *Micromachines* 2022, *13*, 1828

**DOI:** 10.3390/mi16080874

**Published:** 2025-07-29

**Authors:** Rubén Martínez-Revuelta, Horacio I. Solís-Cisneros, Raúl Trejo-Hernández, Madaín Pérez-Patricio, Martha L. Paniagua-Chávez, Rubén Grajales-Coutiño, Jorge L. Camas-Anzueto, Carlos A. Hernández-Gutiérrez

**Affiliations:** 1 Optomechatronics Group, Tecnológico Nacional de México Campus Tuxtla Gutiérrez, Carretera Panamericana Km 1080, Tuxtla Gutiérrez 29050, Mexico; d19270973@tuxtla.tecnm.mx (R.M.-R.); hsolis-cisneros@ieee.org (H.I.S.-C.); madain.pp@tuxtla.tecnm.mx (M.P.-P.); d97270383@tuxtla.tecnm.mx (M.L.P.-C.); ruben.gc@tuxtla.tecnm.mx (R.G.-C.); jcamas@tuxtla.tecnm.mx (J.L.C.-A.); 2 Programa de Nanociencias y Nanotecnología, Centro de Investigación y de Estudios Avanzados del Instituto Politécnico Nacional, Av. Instituto Politécnico Nacional 2508, Ciudad de México 07360, Mexico; raul.hernandez@cinvestav.mx

The journal retracts the article titled “Numerical Study of a Solar Cell to Achieve the Highest InGaN Power Conversion Efficiency for the Whole In-Content Range” [1], cited above.

Following publication, the authors contacted the Editorial Office to raise concerns relating to the scientific accuracy of the data presented in the published paper [1].

Adhering to our standard procedure, the Editorial Office and Editorial Board conducted an investigation that confirmed an error in the calculation of EQE, which fails to account for the drop at the InN bandgap (0.7 eV or 1.77 µm). This oversight results in an erroneous inclusion of wavelength contributions beyond 1.77 µm, leading to an overestimation of both the photocurrent and open-circuit voltage (Voc). Consequently, the model incorrectly assumes that only a few nanometers of material are needed to absorb incoming light, which would leave insufficient material for carrier recombination. This error artificially reduces or eliminates natural recombination, further inflating the calculated efficiency. Since the further findings do not support the central results in the paper, as a result, the Editorial Board and authors have decided to retract this article as per MDPI’s retraction policy (https://www.mdpi.com/ethics#_bookmark30).

This retraction was approved by the Section Editor-in-Chief of the *Micromachines* journal.

The authors agreed to this retraction.
